# Dynamic Behavior of PVC Gel Actuators: Nonlinear Effects of Viscoelasticity and Electromechanical Coupling

**DOI:** 10.3390/polym17050633

**Published:** 2025-02-26

**Authors:** Yang Xiao, Zhigang Chen, Ye Wang, Hanjing Lu, Bin Luo

**Affiliations:** 1 Key Laboratory of Hunan Province for Efficient Power System and Intelligent Manufacturing, College of Mechanical and Energy Engineering, Shaoyang University, Shaoyang 422000, China; xy1998080833@163.com (Y.X.); 4309@hnsyu.edu.cn (Y.W.); lhj943164009@163.com (H.L.); 2 State Key Laboratory for Strength and Vibration of Mechanical Structures, Xi’an Jiaotong University, Xi’an 710049, China

**Keywords:** PVC gel, viscoelasticity, electromechanical constitutive model, Lyapunov exponent, stability

## Abstract

As an inherent property of polyvinyl chloride (PVC) gel material, viscoelasticity is closely related to the deformation of the material, which will affect its dynamic behavior. However, the existing theoretical model does not consider the influence of time-varying damping on its nonlinear vibration, which leads to the unclear nonlinear dynamic behavior of the material under the dual influence of viscoelasticity and electromechanical parameters and limits the further application of the material. Therefore, in this study, the standard linear solid (SLS) model was used to describe the time-varying dynamic change of viscoelasticity of PVC gel, and the electromechanical coupling second-order nonlinear constitutive equation of PVC gel actuator was established by combining the Gent free energy theory model. The harmonic resonance, stability and periodicity of PVC gel actuator under different loading conditions were investigated by using dynamic analysis methods such as phase path, Poincaré map, bifurcation diagram, and Lyapunov exponent. Through the systematic research in this study, the deformation law of PVC gel with time-varying damping under different electromechanical parameters was revealed, and the parameter control strategy of deformation stability and chaos was obtained, which provided the design method and theoretical basis for the further application of the material.

## 1. Introduction

Polyvinyl chloride (PVC) gel is a new type of electroactive polymer material [1,2,3,4,5]. Due to its advantages of low density, low energy consumption, and large deformation, it has broad application prospects in international mechanical frontier disciplines such as artificial muscles and soft robots [6,7,8,9]; for example, bionic gecko toe adhesion desorption devices [10], bionic fish tails [11], and finger tremor suppression orthoses [12], Braille display [13], sensors and actuators [14,15,16,17]. In the practical engineering application of this material, the load applied to the PVC gel material is always periodic voltage or stress [18,19]. The PVC gel exhibits a complex strain–stress relationship and dynamic response, which is necessary for simulating and understanding the vibration of the nonlinear dynamic system of PVC gel [20]. However, the research on the dynamic deformation behavior and electromechanical coupling characteristics of the material under variable load has not received enough attention, resulting in the lack of design methods and theoretical basis, which has limited its further use. Clarifying the relationship between viscoelasticity and vibration behavior is crucial for optimizing the design and control of PVC gel actuators.

For this reason, many scholars have begun to study the theoretical modeling and dynamic deformation behavior of the material from the application. For example, Lei [21] conducted quasi-static and dynamic mechanical compression tests on polyvinyl chloride elastomers under different conditions. Young’s hyperelastic model and the rate-dependent viscoelastic model were established. The model accurately describes the mechanical behavior of polyvinyl chloride. Du [22] established the constraint theory model of the circular plane PVC gel actuator and carried out finite element simulation verification to analyze the vibration characteristics of the actuator. Li [23] used the Gent model to predict the deformation theory of fiber-confined PVC gel, predicted the electrical response before different stretching and voltage amplitude levels, and proved the correctness of the prediction results through experimental tests. From the current research results, the existing theoretical model does not consider the inherent viscoelastic time-varying characteristics of the material, resulting in the theory can not accurately reflect the dynamic response of PVC gel materials. The standard linear solid (SLS) model and the Gent free energy equation [24] can be used to describe the dynamic behavior of PVC gel films with viscoelasticity under the dual effects of viscoelasticity and electromechanical parameters [25,26].

In the following research, this paper deduces the second-order nonlinear differential equation for controlling the deformation of PVC gel film. Through the analysis of the theoretical equation, the influence of key parameters such as shear modulus, applied voltage and prestress on the nonlinear vibration of the actuator under the condition of time-varying damping is systematically studied. The harmonic resonance, stability and periodicity of PVC gel actuator under different operating conditions were investigated by using nonlinear dynamic analysis methods such as phase path, Poincaré map, amplitude–frequency diagram, bifurcation diagram and Lyapunov exponent. Through the systematic research in this study, the vibration law of PVC gel with time-varying damping under different electromechanical parameters was revealed, and the parameter control strategy of its deformation stability and chaotic state was obtained, which provided a theoretical basis for the application of this material in the design of new sensors, vibration absorption, or energy conversion devices.

## 2. Deformation Mechanism and Dynamic Model of PVC Gel Actuator

### 2.1. Deformation Mechanism of Viscoelastic PVC Gel

**Figure 1:**
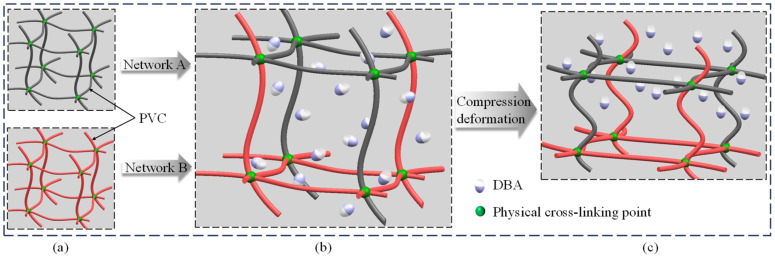
PVC gel deformation mechanism model. (**a**) Polymer skeleton structure model of network A and network B. (**b**) Interlaced binding of polymer backbone space of network A and network B. (**c**) Under the condition of voltage and external force, the polymer skeleton structure of network A and network B is compressed and deformed.

PVC gel is a two-phase material composed of polyvinyl chloride long chain and dibutyl adipate (DBA) molecules. Because it contains both solid polymer lattice and liquid plasticizer, PVC gel is sticky and elastic [27,28,29]. PVC gel is composed of two kinds of PVC long-chain molecular space network structures [30,31,32,33]. As shown in Figure 1a, network A is an ideal hyperelastic PVC network skeleton, network B is a viscoelastic PVC network skeleton, and a physical crosslinking point is formed between the long chains of PVC molecules. Networks A and B are crosslinked and intertwined into the PVC gel space structure skeleton, and the DBA molecules are dispersed in the PVC space network, as shown in Figure 1b. Under the stimulation of AC voltage and pre-stress, DBA molecules undergo directional migration, resulting in compression deformation of the PVC molecular space network, as shown in Figure 1c.

**Figure 2:**
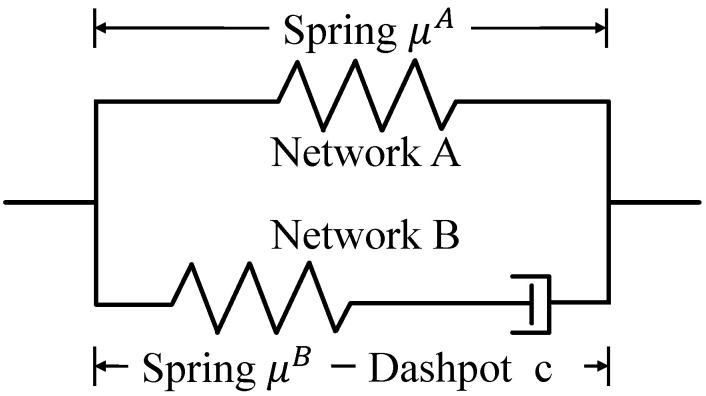
PVC gel material composition network framework: standard linear solid model.

According to the existing research [34,35], the viscoelastic behavior of this two-phase material can be modeled by the generalized Maxwell viscoelastic material model. This paper’s deformation mechanism of time-dependent viscoelastic PVC gel was characterized by the standard linear solid (SLS) model [36,37], as shown in Figure 2. The network A is composed of a superelastic spring with reversible deformation, and the shear modulus is $\mu^{A}$. Network B is composed of a hyperelastic spring with reversible deformation and a damper with irreversible deformation in series, which is used to represent the damping effect of PVC gel. The shear modulus of the spring is $\mu^{B}$, and the viscous damping coefficient of the damper is c. Finally, network A and network B are connected in parallel to form the overall structure of PVC gel. We set the deformation of the spring in network A as λ, the deformation of the spring in network B as $\lambda_{e}$, and the deformation of the damper as ξ. In this model, the deformation relationship between the spring and the damper is $\xi =\lambda /\lambda_{e}$.

### 2.2. Dynamic Model of PVC Gel Actuator

**Figure 3:**
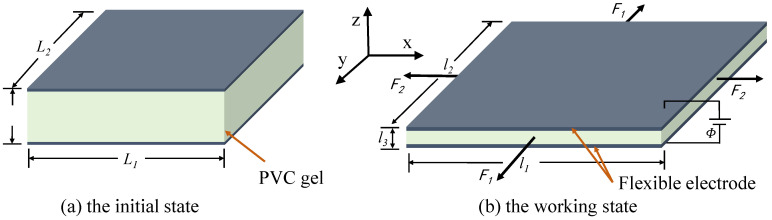
Simple physical model of PVC gel actuator. (**a**) In the initial state, the sizes of PVC gel actuators in the x, y, and z directions are L_1_, L_2_, and L_3_, respectively. (**b**) In the working state, the PVC gel actuator is subjected to prestress F_1_ and F_2_, and the voltage AA, and the dimensions are changed to l_1_, l_2_, and l_3_, respectively.

The simple physical model of the PVC gel actuator is shown in Figure 3. Figure 3a is a PVC gel actuator in the initial state. The upper and lower layers are conductive flexible electrodes, and the middle is a PVC gel film, forming a “Sandwich” structure. At this time, the dimensions of the PVC gel actuator in the x, y, and z directions are denoted as L_1_, L_2_ and L_3_, respectively. After applying prestressing F_1_ and F_2_ and AC voltage *φ*, the PVC gel actuator undergoes in-plane deformation; the thickness decreases, the area increases, and changes into the working state shown in Figure 3b. At this time, the sizes in the x, y and z directions increase to l_1_, l_2_ and l_3_, respectively. Therefore, we defined the deformation in the x, y and z directions as $\lambda_{1}=l_{1}/L_{1}$, $\lambda_{2}=l_{2}/L_{2}$ and $\lambda_{3}=l_{3}/L_{3}$. The thickness of the PVC gel film is 1 mm, the side length is 40 mm square, and the thickness of the upper and lower electrodes is 0.1 mm.

PVC gel is a kind of superelastic and high-strain material. We use the Gent free energy model to describe the energy change of PVC gel. The Gent model is a constitutive model for describing hyperelastic materials, which is especially suitable for rubber-like materials with large deformation characteristics. The unit free energy density of PVC gel is:

(1) $$\begin{array}{l} W=-\frac{\mu^{A}J_{m}^{A}}{2}\log\left(1-\frac{\lambda_{1}^{2}+\lambda_{2}^{2}+\lambda_{1}^{-2}\lambda_{2}^{-2}-3}{J_{m}^{A}}\right)\\ -\frac{\mu^{B}J_{m}^{B}}{2}\log\left(1-\frac{\lambda_{1}^{2}\xi_{1}^{-2}+\lambda_{2}^{2}\xi_{2}^{-2}+\lambda_{1}^{-2}\lambda_{2}^{-2}\xi_{1}^{2}\xi_{2}^{2}-3}{J_{m}^{B}}\right)+\frac{D^{2}}{2\varepsilon } \end{array}$$

In the formula, W is the free energy per unit volume, and the unit is $J\cdot m^{-3}$. $J_{m}^{A}$ and $J_{m}^{B}$ are the constants of the tensile limit of the molecular chain of the hyperelastic material of the A and B springs in the SLS model, respectively. $\mu^{A}$ and $\mu^{B}$ are the shear modulus of spring A and spring B, respectively, and the unit is Pa. D is the real electric displacement. Under the action of AC voltage on the PVC gel film, the relationship between the real electric displacement D and the real electric field strength E is D=εE, where ε is the dielectric constant of the PVC gel and the electric field strength $E=\Phi /L_{3}\lambda_{3}$. As the Poisson’s ratio of PVC gel is close to the incompressible condition, $\lambda_{1}\lambda_{2}\lambda_{3}=1$ is obtained.

Since the flexible actuator is a thin film structure, the thickness in the z direction is negligible. Therefore, only the inertial forces of the PVC gel actuator in the x and y directions are $\rho L_{2}L_{3}x^{2}\left(d^{2}\lambda_{1}/dt^{2}\right)$ and $\rho L_{1}L_{3}y^{2}\left(d^{2}\lambda_{2}/dt^{2}\right)$, respectively, and the damping forces in the x and y directions are $cxd\xi_{1}/dt$ and $cyd\xi_{2}/dt$, respectively, where c is the viscous damping coefficient, and the unit is N⋅s/m.

The work conducted by the inertial force and damping force in the two directions can be obtained by integrating the inertial force and damping force in the x and y directions, respectively; that is:

(2) $$\rho L_{2}L_{3}\delta \lambda_{1}\frac{d^{2}\lambda_{1}}{dt^{2}}\int_{0}^{L_{1}}x^{2}dx=\frac{\rho L_{1}^{3}L_{2}L_{3}}{3}\delta \lambda_{1}\frac{d^{2}\lambda_{1}}{dt^{2}}$$

(3) $$\rho L_{1}L_{3}\delta \lambda_{2}\frac{d^{2}\lambda_{2}}{dt^{2}}\int_{0}^{L_{2}}y^{2}dy=\frac{\rho L_{2}^{3}L_{1}L_{3}}{3}\delta \lambda_{2}\frac{d^{2}\lambda_{2}}{dt^{2}}$$

(4) $$c\delta \xi_{1}\frac{d\xi_{1}}{dt}\int_{0}^{L_{1}}xdx=\frac{cL_{1}^{2}}{2}\delta \xi_{1}\frac{d\xi_{1}}{dt}$$

(5) $$c\delta \xi_{2}\frac{d\xi_{2}}{dt}\int_{0}^{L_{2}}ydy=\frac{cL_{2}^{2}}{2}\delta \xi_{2}\frac{d\xi_{2}}{dt}$$

Through the principle of energy conservation, the free energy change of the PVC gel actuator is equal to the sum of voltage, prestress, inertia force, damping force, and work. The equation can be obtained as follows:

(6) $$\begin{array}{ll} L_{1}L_{2}L_{3}\delta W & =\phi \delta Q+F_{1}L_{1}\delta \lambda_{1}+F_{2}L_{2}\delta \lambda_{2}\\ & -\frac{L_{1}^{3}\rho L_{2}L_{3}}{3}\delta \lambda_{1}\frac{d^{2}\lambda_{1}}{dt^{2}}-\frac{L_{2}^{3}\rho L_{1}L_{3}}{3}\delta \lambda_{2}\frac{d^{2}\lambda_{2}}{dt^{2}}\\ & -\frac{cL_{1}^{2}}{2}\delta \xi_{1}\frac{d\xi_{1}}{dt}-\frac{cL_{2}^{2}}{2}\delta \xi_{2}\frac{d\xi_{2}}{dt} \end{array}$$

According to Equation (6), the derivatives of $\lambda_{1},\lambda_{2},\xi_{1},\xi_{2}\;\mathrm{and}\;D$ can be obtained respectively:

(7) $$\frac{\partial W}{\partial \lambda_{1}}=\frac{\phi D}{L_{3}}\lambda_{2}+\frac{F_{1}}{L_{2}L_{3}}-\frac{\rho L_{1}^{2}}{3}\frac{d^{2}\lambda_{1}}{dt^{2}}$$

(8) $$\frac{\partial W}{\partial \lambda_{2}}=\frac{\phi D}{L_{3}}\lambda_{1}+\frac{F_{2}}{L_{1}L_{3}}-\frac{\rho L_{2}^{2}}{3}\frac{d^{2}\lambda_{2}}{dt^{2}}$$

(9) $$\frac{\partial W}{\partial \xi_{1}}=\frac{cL_{1}}{2L_{2}L_{3}}\frac{d\xi_{1}}{dt}$$

(10) $$\frac{\partial W}{\partial \xi_{2}}=\frac{cL_{2}}{2L_{1}L_{3}}\frac{d\xi_{2}}{dt}$$

(11) $$\frac{\partial W}{\partial D}=\frac{\phi \lambda_{1}\lambda_{2}}{L_{3}}$$

The partial derivative of Equation (1) can be obtained as follows:

(12) $$\begin{array}{ll} \frac{\partial W}{\partial \lambda_{1}}= & \frac{\mu^{A}\left(\lambda_{1}-\lambda_{1}^{-3}\lambda_{2}^{-2}\right)}{1-\left(\lambda_{1}^{2}+\lambda_{2}^{2}+\lambda_{1}^{-2}\lambda_{2}^{-2}-3\right)/J_{m}^{A}}\\ & +\frac{\mu^{B}\left(\lambda_{1}\xi_{1}^{-2}-\lambda_{1}^{-3}\lambda_{2}^{-2}\xi_{1}^{2}\xi_{2}^{2}\right)}{1-\left(\lambda_{1}^{2}\xi_{1}^{-2}+\lambda_{2}^{2}\xi_{2}^{-2}+\lambda_{1}^{-2}\lambda_{2}^{-2}\xi_{1}^{2}\xi_{2}^{2}-3\right)/J_{m}^{B}} \end{array}$$

(13) $$\begin{array}{ll} \frac{\partial W}{\partial \lambda_{2}}= & \frac{\mu^{A}\left(\lambda_{2}-\lambda_{2}^{-3}\lambda_{1}^{-2}\right)}{1-\left(\lambda_{1}^{2}+\lambda_{2}^{2}+\lambda_{1}^{-2}\lambda_{2}^{-2}-3\right)/J_{m}^{A}}\\ & +\frac{\mu^{B}\left(\lambda_{2}\xi_{2}^{-2}-\lambda_{2}^{-3}\lambda_{1}^{-2}\xi_{1}^{2}\xi_{2}^{2}\right)}{1-\left(\lambda_{1}^{2}\xi_{1}^{-2}+\lambda_{2}^{2}\xi_{2}^{-2}+\lambda_{1}^{-2}\lambda_{2}^{-2}\xi_{1}^{2}\xi_{2}^{2}-3\right)/J_{m}^{B}} \end{array}$$

(14) $$\frac{\partial W}{\partial \xi_{1}}=-\frac{\mu^{B}\left(\lambda_{1}^{2}\xi_{1}^{-3}-\lambda_{1}^{-2}\lambda_{2}^{-2}\xi_{1}\xi_{2}^{2}\right)}{1-\left(\lambda_{1}^{2}\xi_{1}^{-2}+\lambda_{2}^{2}\xi_{2}^{-2}+\lambda_{1}^{-2}\lambda_{2}^{-2}\xi_{1}^{2}\xi_{2}^{2}-3\right)/J_{m}^{B}}$$

(15) $$\frac{\partial W}{\partial \xi_{2}}=-\frac{\mu^{B}\left(\lambda_{2}^{2}\xi_{2}^{-3}-\lambda_{1}^{-2}\lambda_{2}^{-2}\xi_{1}^{2}\xi_{2}\right)}{1-\left(\lambda_{1}^{2}\xi_{1}^{-2}+\lambda_{2}^{2}\xi_{2}^{-2}+\lambda_{1}^{-2}\lambda_{2}^{-2}\xi_{1}^{2}\xi_{2}^{2}-3\right)/J_{m}^{B}}$$

(16) $$\frac{\partial W}{\partial D}=\frac{D}{\varepsilon }$$

By combing and integrating the above Equations (7)–(16) and eliminating the real electric displacement D contained in the equation, the deformation control equations of PVC gel in the x and y directions and the deformation control equations of the damper can be obtained as follows:

(17) $$\begin{array}{l} \frac{\rho L_{1}^{2}}{3\mu^{A}}\frac{d^{2}\lambda_{1}}{dt^{2}}-\frac{\varepsilon \phi^{2}}{\mu^{A}L_{3}^{2}}\lambda_{1}\lambda_{2}^{2}-\frac{F_{1}}{\mu^{A}L_{2}L_{3}}+\frac{\left(\lambda_{1}-\lambda_{1}^{-3}\lambda_{2}^{-2}\right)}{1-\left(\lambda_{1}^{2}+\lambda_{2}^{2}+\lambda_{1}^{-2}\lambda_{2}^{-2}-3\right)/J_{m}^{A}}\\ +\frac{\mu^{B}}{\mu^{A}}\frac{\left(\lambda_{1}\xi_{1}^{-2}-\lambda_{1}^{-3}\lambda_{2}^{-2}\xi_{1}^{2}\xi_{2}^{2}\right)}{1-\left(\lambda_{1}^{2}\xi_{1}^{-2}+\lambda_{2}^{2}\xi_{2}^{-2}+\lambda_{1}^{-2}\lambda_{2}^{-2}\xi_{1}^{2}\xi_{2}^{2}-3\right)/J_{m}^{B}}=0 \end{array}$$

(18) $$\begin{array}{l} \frac{\rho L_{2}^{2}}{3\mu^{A}}\frac{d^{2}\lambda_{2}}{dt^{2}}-\frac{\varepsilon \phi^{2}}{\mu^{A}L_{3}^{2}}\lambda_{2}\lambda_{1}^{2}-\frac{F_{2}}{\mu^{A}L_{1}L_{3}}+\frac{\left(\lambda_{2}-\lambda_{2}^{-3}\lambda_{1}^{-2}\right)}{1-\left(\lambda_{1}^{2}+\lambda_{2}^{2}+\lambda_{1}^{-2}\lambda_{2}^{-2}-3\right)/J_{m}^{A}}\\ +\frac{\mu^{B}}{\mu^{A}}\frac{\mu^{B}\left(\lambda_{2}\xi_{2}^{-2}-\lambda_{2}^{-3}\lambda_{1}^{-2}\xi_{1}^{2}\xi_{2}^{2}\right)}{1-\left(\lambda_{1}^{2}\xi_{1}^{-2}+\lambda_{2}^{2}\xi_{2}^{-2}+\lambda_{1}^{-2}\lambda_{2}^{-2}\xi_{1}^{2}\xi_{2}^{2}-3\right)/J_{m}^{B}}=0 \end{array}$$

(19) $$\frac{d\xi_{1}}{dt}=\frac{2L_{2}L_{3}\mu^{B}}{cL_{1}}\frac{\left(\lambda_{1}^{2}\xi_{1}^{-3}-\lambda_{1}^{-2}\lambda_{2}^{-2}\xi_{1}\xi_{2}^{2}\right)}{1-\left(\lambda_{1}^{2}\xi_{1}^{-2}+\lambda_{2}^{2}\xi_{2}^{-2}+\lambda_{1}^{-2}\lambda_{2}^{-2}\xi_{1}^{2}\xi_{2}^{2}-3\right)/J_{m}^{B}}$$

(20) $$\frac{d\xi_{2}}{dt}=\frac{2L_{1}L_{3}\mu^{B}}{cL_{2}}\frac{\left(\lambda_{2}^{2}\xi_{2}^{-3}-\lambda_{1}^{-2}\lambda_{2}^{-2}\xi_{1}^{2}\xi_{2}\right)}{1-\left(\lambda_{1}^{2}\xi_{1}^{-2}+\lambda_{2}^{2}\xi_{2}^{-2}+\lambda_{1}^{-2}\lambda_{2}^{-2}\xi_{1}^{2}\xi_{2}^{2}-3\right)/J_{m}^{B}}$$

In order to analyze the deformation of PVC gel, the force-electric coupling dynamics of square PVC gel actuator is studied, so the relevant conditions are simplified. The length and width of the film in the x and y directions are equal, denoted as $L_{1}=L_{2}=L$; the deformations in the two directions are equal, denoted as $\lambda_{1}=\lambda_{2}=\lambda$; the prestresses in the two directions are equal, denoted as $\bar{F}=F_{1}/\mu^{A}L_{2}L_{3}=F_{2}/\mu^{A}L_{1}L_{3}$. Simplifying Equations (17)–(20), we can obtain:

(21) $$\begin{array}{l} \frac{d^{2}\lambda }{dT^{2}}-\Phi^{2}\lambda^{3}-\bar{F}+\frac{\left(\lambda -\lambda^{-5}\right)}{1-\left(2\lambda^{2}+\lambda^{-4}-3\right)/J_{m}}\\ +\frac{\mu^{B}}{\mu^{A}}\frac{\left(\lambda \xi^{-2}-\lambda^{-5}\xi^{4}\right)}{1-\left(2\lambda^{2}\xi^{-2}+\lambda^{-4}\xi^{4}-3\right)/J_{m}}=0. \end{array}$$

(22) $$\frac{d\xi }{dT}=\frac{1}{C_{d}}\frac{\left(\lambda^{2}\xi^{-3}-\lambda^{-4}\xi^{3}\right)}{1-\left(2\lambda^{2}\xi^{-2}+\lambda^{-4}\xi^{4}-3\right)/J_{m}}$$

Equations (21) and (22) are the expressions of the relationship between the deformation of PVC gel and the deformation of damper and time *T* in the model. In order to facilitate the understanding and discussion of PVC gel deformation, we now perform dimensionless simplification of the relevant parameters in the equation. Among them, $T=t/\sqrt{\rho L_{1}^{2}/3\mu^{A}}=t/\sqrt{\rho L_{2}^{2}/3\mu^{A}}$ is the dimensionless time, $\Phi =\left(\varepsilon \phi^{2}\right)/\left(\mu^{A}L_{3}^{2}\right)$ is the dimensionless voltage; $C_{d}=\left(cL_{1}\right)/\left(2L_{2}L_{3}\mu^{B}\sqrt{\rho L_{1}^{2}/3\mu^{A}}\right)=\left(cL_{2}\right)/\left(2L_{1}L_{3}\mu^{B}\sqrt{\rho L_{2}^{2}/3\mu^{A}}\right)$ is the dimensionless viscous damping coefficient. The Poincaré map is a mapping defined by the trajectory motion in phase space. When the trajectory repeatedly crosses the same section, it reflects the mapping of the dependence of the subsequent point on the previous point. According to the situation that the trajectory passes through the section, the shape of the motion can be judged concisely. In the following, the nonlinear dynamic characteristics of the PVC gel actuator system will be quantitatively analyzed by comparing the time-domain vibration displacement response, phase paths, Poincaré maps, and Lyapunov exponent of different viscous damping coefficients.

## 3. Dynamic Characteristics Analysis of PVC Gel Actuator Under Different Parameters

In this study, the sinusoidal voltage applied to the upper and lower electrodes of PVC gel is $\Phi =\Phi_{0}\sin(\omega T)$, where $\Phi_{0}$ is the dimensionless voltage amplitude and ω is the dimensionless frequency. Under this AC voltage and action, the change of different parameters will make the vibration response of PVC gel show different trends. Let the initial parameter viscous damping coefficient $C_{d}=0.01$, the ratio of shear modulus $\mu_{B}/\mu_{A}=1$, the voltage amplitude $\Phi_{0}=0.2$, and the prestress $\bar{F}=0.2$. In order to facilitate the calculation, the ultimate tensile parameter $J_{m}=60$ is set.

**Figure 4:**
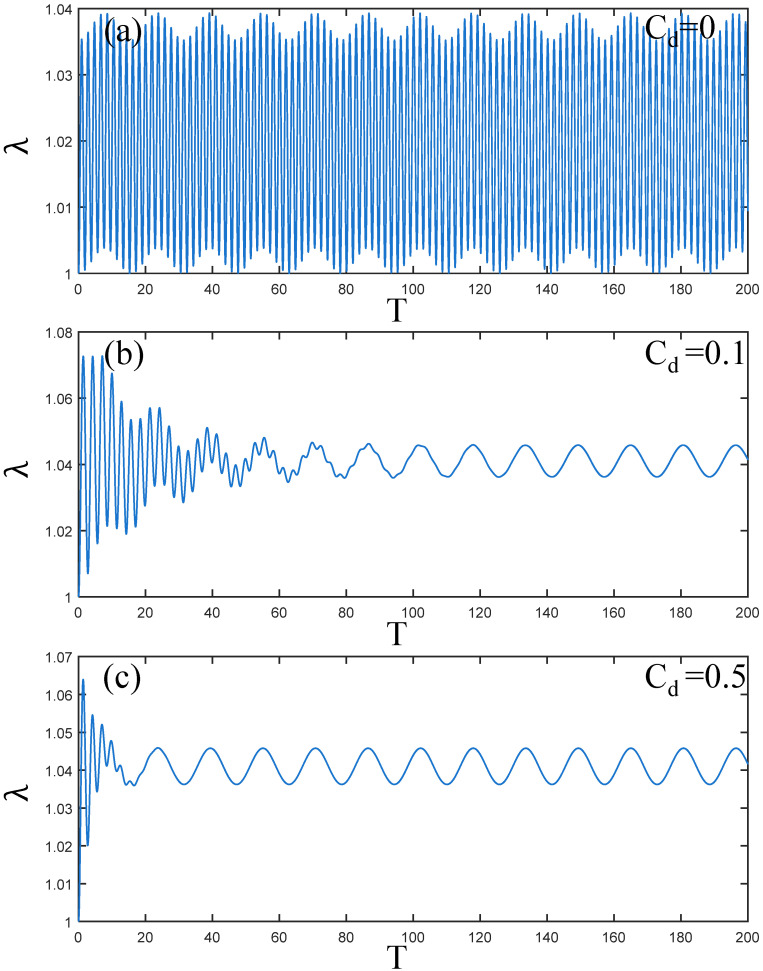
Under the condition of shear modulus ratio $\mu_{B}/\mu_{A}=1$, voltage amplitude $\Phi_{0}=0.2$, and prestress $\bar{F}=0.2$, (**a**–**c**) is the dynamic response when $C_{d}=0,0.1\;\mathrm{and}\;0.5$. The greater the viscous damping coefficient, the faster the amplitude attenuation of PVC gel film.

According to the damping PVC gel actuator studied by Li [38], the viscous damping coefficient ranges from 0 to 0.5. Therefore, as shown in Figure 4a–c, the amplitude changes in the PVC gel when the viscous damping coefficient $C_{d}=0,\;0.1\;\mathrm{and}\;0.5$. When $C_{d}=0$, the amplitude of the PVC gel actuator is stable, showing a stable periodic change. With the increase in damping, when the viscous damping coefficient $C_{d}=0.1$, the amplitude of PVC gel in the initial time region is larger. When 40≤T≤100, PVC gel has a small perturbation vibration under the overall vibration condition. After T=100, the amplitude of the PVC gel actuator tends to be stable. Finally, when $C_{d}=0.5$, the PVC gel quickly enters a stable periodic vibration at T=20 in Figure 4c. Therefore, the amplitude of the PVC gel actuator is obviously affected by the damping effect. With the increase in damping, the amplitude of PVC gel decreases with the increase in time *T* and tends to be stable in terms of periodic vibration.

**Figure 5:**
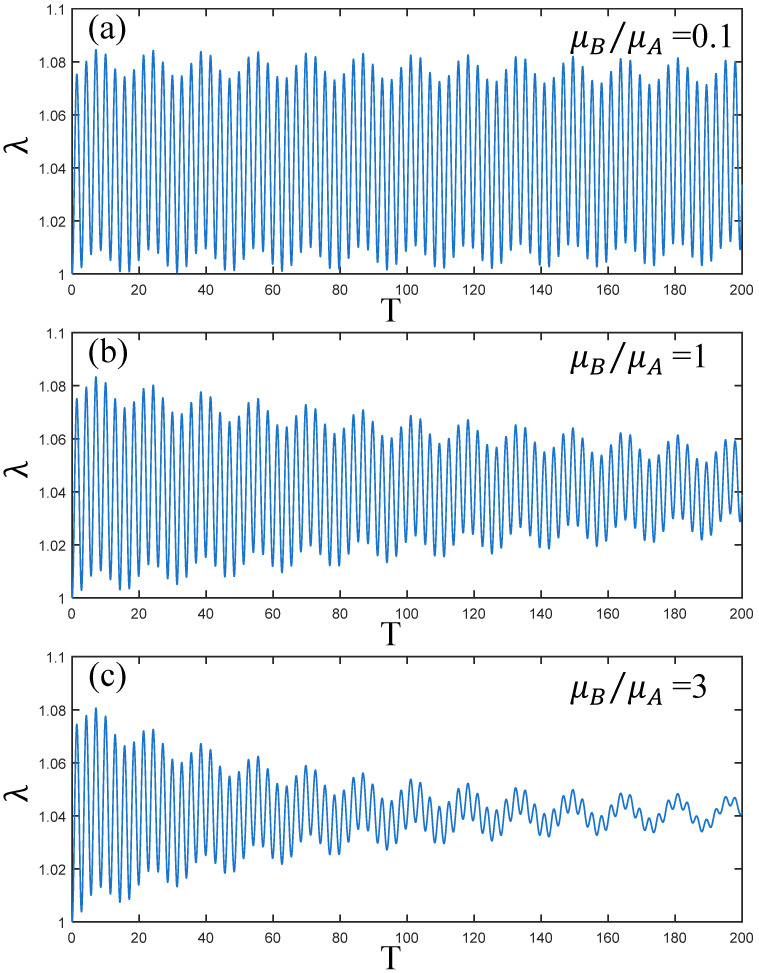
Under the condition of viscous damping coefficient $C_{d}=0.01$, voltage amplitude $\Phi_{0}=0.2$, prestress $\bar{F}=0.2$, (**a**–**c**) is the dynamic response when $\mu_{B}/\mu_{A}=0.1,1\;\mathrm{and}\;3$. The larger the ratio of shear modulus, the weaker the amplitude of PVC gel film.

Figure 5 reflects the vibration state of the PVC gel actuator when the ratio of shear modulus $\mu_{B}/\mu_{A}=0.1,\;1\;\mathrm{and}\;3$. Comparing Figure 5a–c, it can be seen that the initial amplitude of the PVC gel actuator is the same under different $\mu_{B}/\mu_{A}$ conditions. Due to the existence of damping, with the increase $\mu_{B}/\mu_{A}$, the vibration of the PVC gel actuator will attenuate continuously under the change of time. The larger the $\mu_{B}/\mu_{A}$, the lower the amplitude and the faster the attenuation speed. Therefore, it can be seen that the larger $\mu_{B}/\mu_{A}$ makes the vibration of the PVC gel actuator more stable. However, it can be seen from Figure 5c that small disturbances occur while the amplitude is weakened.

**Figure 6:**
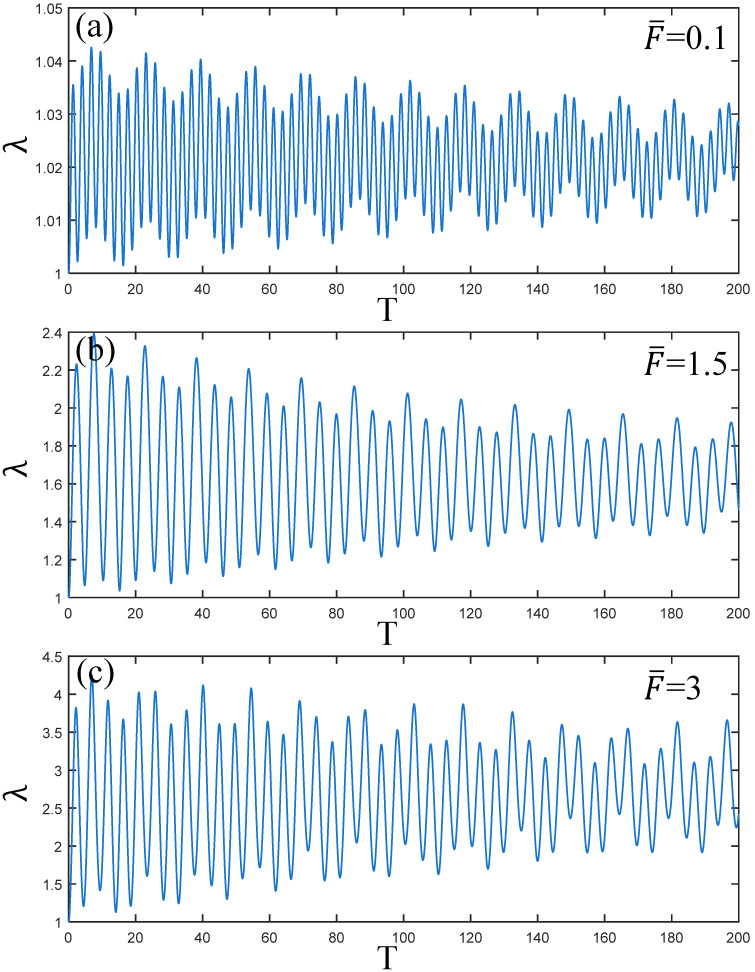
Under the condition of viscous damping coefficient $C_{d}=0.01$, voltage amplitude $\Phi_{0}=0.2$, prestress $\bar{F}=0.2$, (**a**–**c**) is the dynamic response when $\mu_{B}/\mu_{A}=0.1,1.5\;\mathrm{and}\;3$. The larger the ratio of shear modulus, the weaker the amplitude of PVC gel film.

Figure 6 illustrates the change in amplitude of the PVC gel actuator as the pre-stress $\bar{F}$ changes. From the comparison of Figure 6a–c, it is observed that when $\bar{F}=0.1$ the initial amplitude of PVC gel is low, the vibration frequency is high, while there is a vibration attenuation trend. With the increase in $\bar{F}$, when $\bar{F}$ is 1.5 and 3, the initial amplitude of the PVC gel actuator increases gradually, the vibration frequency decreases continuously, and the attenuation trend of vibration remains unchanged. Therefore, increasing the prestress $\bar{F}$ increases the amplitude of the PVC gel actuator and reduces its vibration frequency. However, the attenuation rate of vibration remains constant regardless of the level of prestress $\bar{F}$.

**Figure 7:**
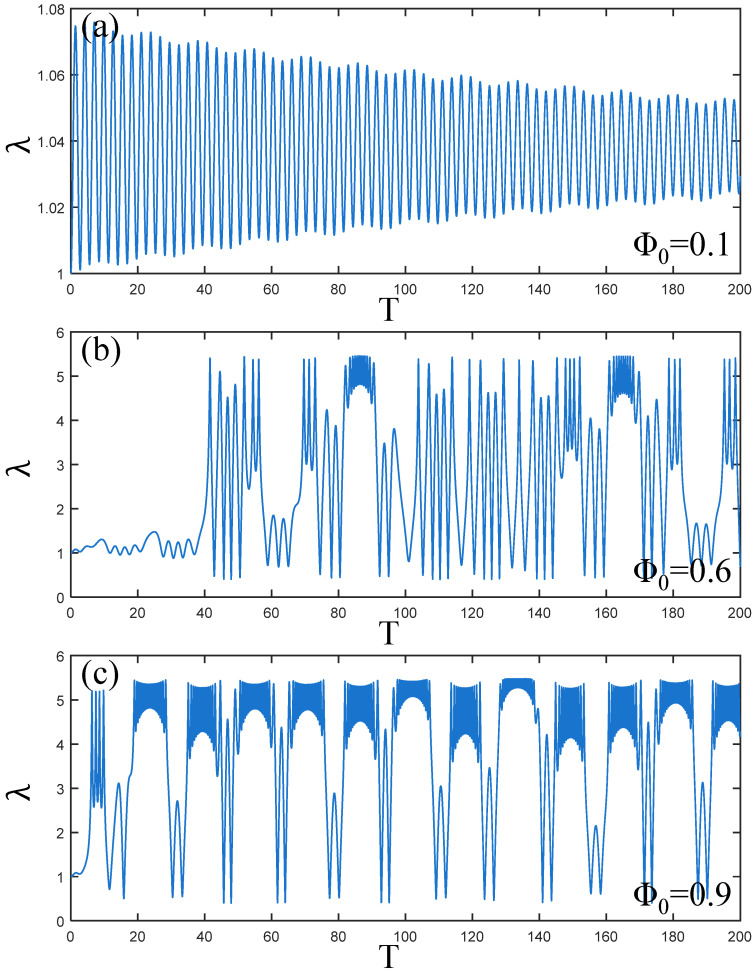
Under the condition of viscous damping coefficient $C_{d}=0.01$, shear modulus ratio $\mu_{B}/\mu_{A}=1$, prestress $\bar{F}=0.2$, (**a**–**c**) is the dynamic response when the voltage amplitude $\Phi_{0}=0.1,0.6,0.9$. The larger the voltage, the larger the amplitude of the PVC gel film, and the vibration is chaotic.

Figure 7a–c illustrate the amplitude changes of the PVC gel actuator during the increase in voltage amplitude $\Phi_{0}$. When $\Phi_{0}=0.1$, the vibration of PVC gel actuator decays uniformly over time. When $\Phi_{0}=0.6$, with the change of time, when T = 40, the PVC gel actuator exhibits violent vibration, reaching maximum amplitude. When $\Phi_{0}=0.9$, the amplitude quickly peaks at the beginning stage. Therefore, it can be seen that the increase in voltage will cause uncontrollable vibration deformation of the PVC gel actuator. The PVC gel actuator is more suitable for low-pressure driving conditions.

## 4. The Stability and Periodicity of PVC Gel Actuator Under Different Parameters

According to the phase paths and Poincaré maps, the PVC gel actuator’s stability and periodic change under single condition change are understood. Let the initial parameters be the viscous damping coefficient $C_{d}=0.01$, the ratio of shear modulus $\mu_{B}/\mu_{A}=1$, the voltage amplitude $\Phi_{0}=0.2$, and the prestress $\bar{F}=0.2$.

**Figure 8:**
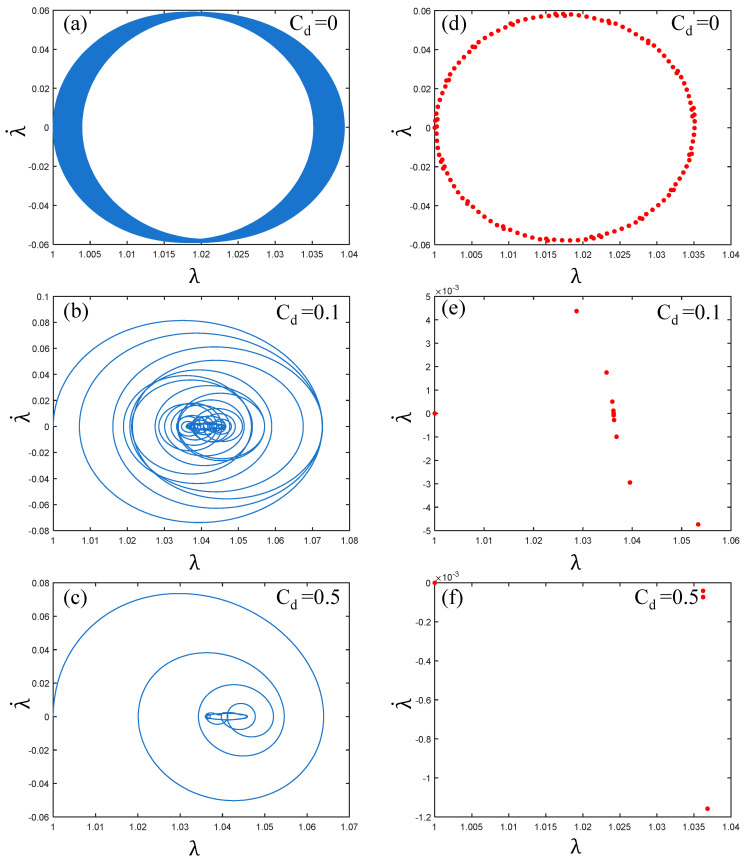
Under the conditions of shear modulus ratio $\mu_{B}/\mu_{A}=1$, voltage amplitude $\Phi_{0}=0.2$ and prestress $\bar{F}=0.2$, when the viscous damping coefficient $C_{d}=0,0.1\;\mathrm{and}\;0.5$, (**a**–**c**) is the phase paths and (**d**–**f**) is the Poincaré maps. The greater the viscous damping coefficient, the more stable the vibration of PVC gel film.

Figure 8 shows the phase path and Poincare map of the PVC gel actuator as the damping coefficient increases. As shown in Figure 8a–c, with the viscous damping coefficient increasing from 0 to 0.1 and then to 0.5, the phase path transitions from a “high-density aggregation ring” to a “spiral” line with a clear trajectory, and the internal center path shows local disorder. Therefore, this indicates that the vibration of the PVC gel actuator gradually becomes stable with the increase in damping yet retains some instability. In Figure 8d–f, the Poincaré map is transformed from a “ring” to an “S-like” shape and finally into an infinite number of points gathered together. It can be seen that the periodicity is getting stronger. In summary, the increase in damping makes the vibration of the PVC gel actuator gradually stable and periodically enhanced.

**Figure 9:**
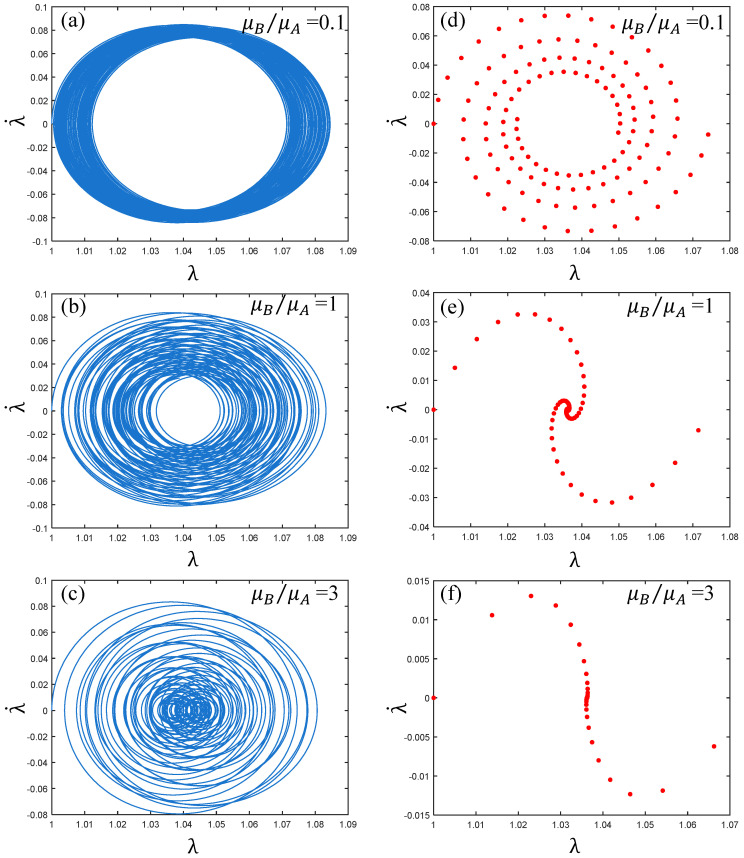
Under the conditions of viscous damping coefficient $C_{d}=0.01$, the voltage amplitude $\Phi_{0}=0.2$, prestress $\bar{F}=0.2$, when the ratio of shear modulus $\mu_{B}/\mu_{A}=0.1,\;1\;\mathrm{and}\;3$, (**a**–**c**) are the phase paths and (**d**–**f**) are the Poincaré maps. The greater the ratio of shear modulus, the more stable the vibration of PVC gel film.

As shown in Figure 9, with the change of the ratio of shear modulus $\mu_{B}/\mu_{A}$, the corresponding phase paths and Poincaré maps state are also different. It can be seen from Figure 9a–c that when the shear modulus $\mu_{B}/\mu_{A}$ increases from 0.1 to 3, the phase paths change from “dense ring” to “plane pie”, but the corresponding coordinate values do not change significantly. Therefore, it can be seen that the vibration of the PVC gel actuator is always in a stable state during the change of shear modulus ratio. It can be seen from Figure 9d–f that the trajectory of Poincaré maps is always clear, so the vibration of the PVC gel actuator is always in a multi-period state without chaos. In summary, the PVC gel actuator has been in a stable multi-period motion state during the change of the shear modulus ratio.

**Figure 10:**
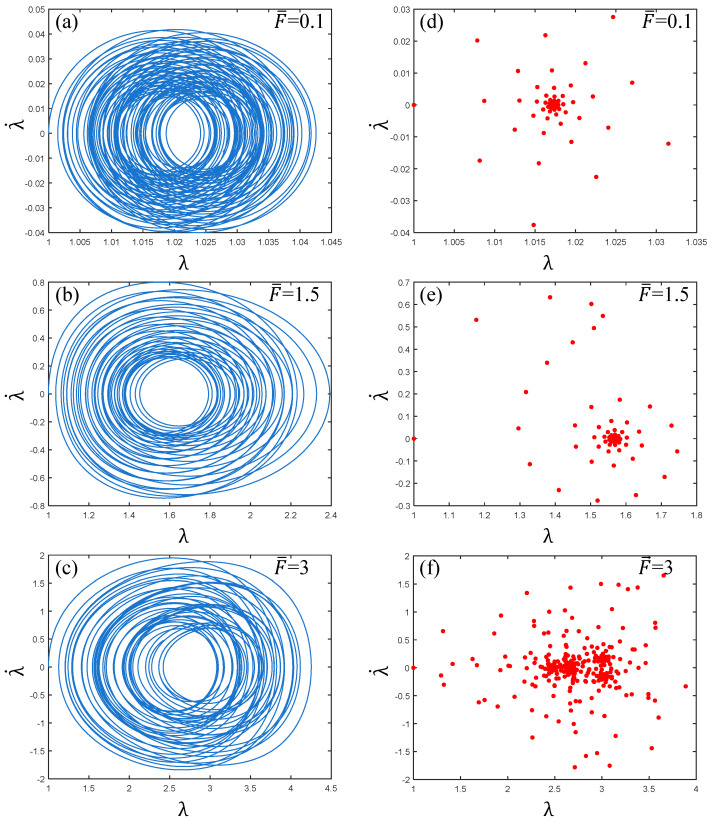
Under the conditions of viscous damping coefficient $C_{d}=0.01$, shear modulus ratio $\mu_{B}/\mu_{A}=1$, and voltage amplitude $\Phi_{0}=0.2$, when the prestress $\bar{F}=0.1,1.5\;\mathrm{and}\;3$, (**a**–c) are the phase paths and (**d**–**f**) are the Poincaré maps. The greater the prestress, the more dispersed the vibration of PVC gel film.

Figure 10 shows the phase paths and Poincaré maps of PVC gel actuator vibration as the prestress changes. It can be seen from Figure 10a–c that with the increase in prestress, the phase path expands when the prestress $\bar{F}=3$, and the coordinate value increases greatly, indicating that the vibration of the PVC gel actuator is unstable with the increase in prestress. Correspondingly, from the Poincaré maps of Figure 10d–f, it can be seen that when $\bar{F}=0.1$, the pattern is “radial”, when $\bar{F}=1.5$, it is “spiral”, when $\bar{F}=3$, the Poincaré map is transformed into a scattered distribution. In summary, when the prestress $\bar{F}=3$, the PVC gel actuator has an unstable, chaotic vibration.

**Figure 11:**
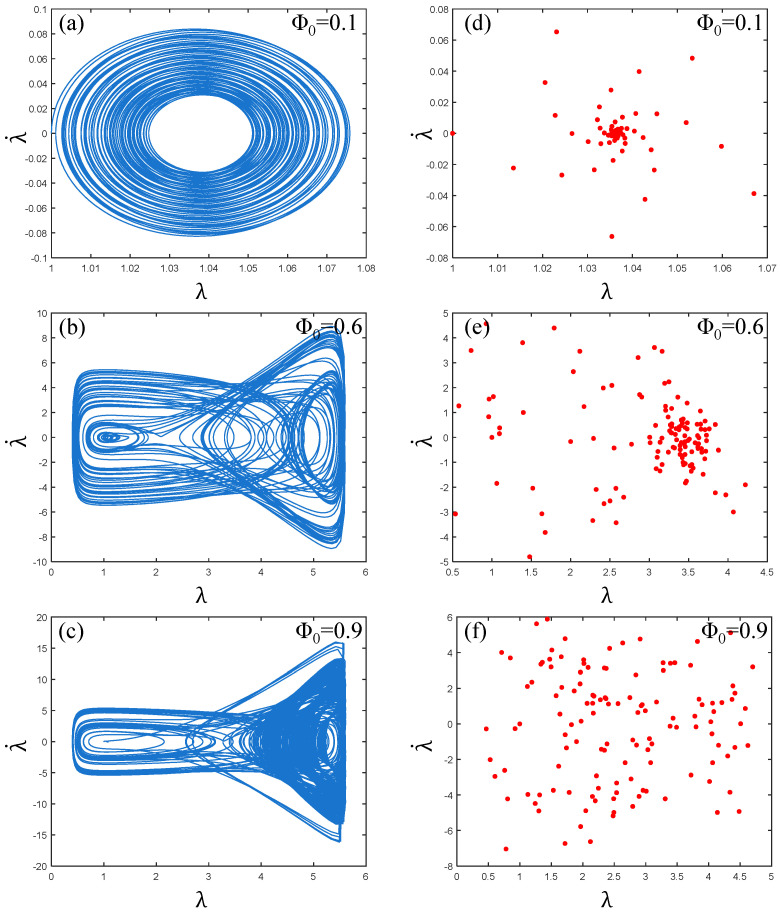
Under the conditions of viscous damping coefficient $C_{d}=0.01$, shear modulus ratio $\mu_{B}/\mu_{A}=1$, prestress $\bar{F}=0.2$, and when the voltage amplitude $\Phi_{0}=0.1,0.6\;\mathrm{and}\;0.9$, (**a**–**c**) are the phase paths and (**d**–**f**) are the Poincaré maps. The larger the voltage amplitude, the more chaotic the vibration of PVC gel film.

It can be seen from Figure 11a,d that the phase path of the PVC gel actuator is clear under low pressure $\Phi_{0}=0.1$, and the Poincaré map pattern is “rotating”. It can be seen that the PVC gel actuator is stable at this time. Multi-period motion. As shown in Figure 11b, when the voltage $\Phi_{0}=0.6$, the phase path trajectory, is in a chaotic crossover state and $\overset{\cdot }{\lambda }$ is in the (−9, 9) interval at this time, the PVC gel actuator is in a completely unstable state. In Figure 11e, the Poincaré map appears scattered without a discernible trajectory, illustrating the chaotic motion of the PVC gel.

Furthermore, as voltage continues to rise $\Phi_{0}=0.9$, the phase path in Figure 11c is more chaotic, and the $\overset{\cdot }{\lambda }$ interval increases to (−18, 18). The points in the Poincaré map of Figure 11f increase and become more scattered, which is in a more chaotic motion state. Therefore, it can be seen that with the increase in voltage, the vibration of the PVC gel actuator changes from stable multi-period vibration to unstable, chaotic vibration, and with the increase in voltage amplitude, the degree of chaos is more intense.

## 5. Resonance Analysis of PVC Gel Actuator

**Figure 12:**
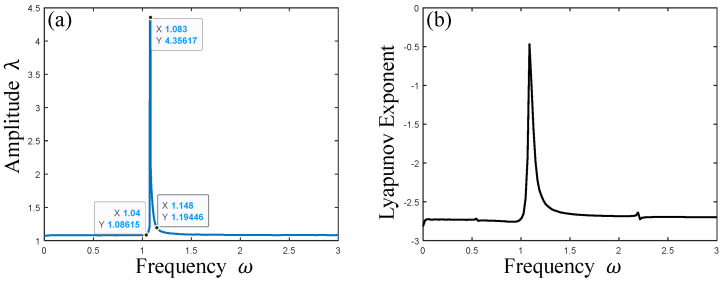
Amplitude-frequency response (**a**) and Lyapunov exponent (**b**) of PVC gel actuator when harmonic resonance occurs.

The preceding discussion explores the amplitude, stability and periodic changes of PVC gel under various factors. In this section, starting from the amplitude–frequency characteristics, the resonance characteristics of PVC gel actuators under various factors are discussed. The viscous damping coefficient $C_{d}=0.01$, the ratio of shear modulus $\mu_{B}/\mu_{A}=1$, the voltage amplitude $\Phi_{0}=0.2$, and the prestress $\bar{F}=0.2$ are still set as the initial parameters. Figure 12 shows the amplitude–frequency response of the PVC gel actuator.

**Figure 13:**
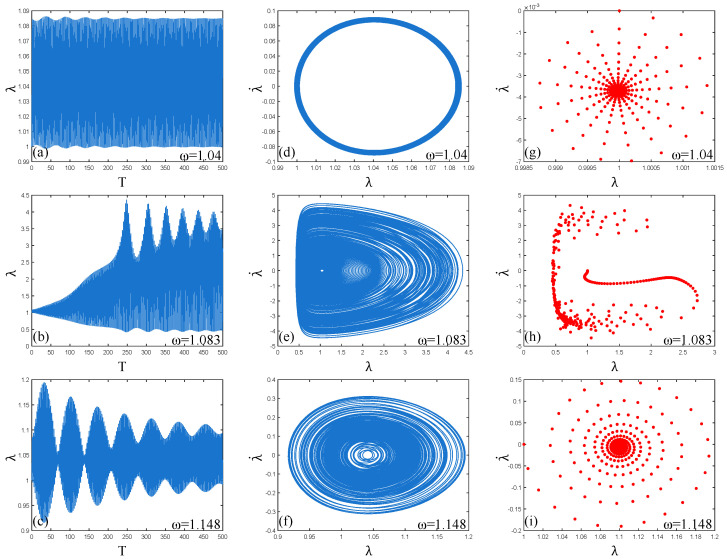
In the harmonic resonance region (1.04–1.148), (**a**–**c**), (**d**–**f**), and (**g**–**i**) are the dynamic response, phase paths, and Poincaré maps of the PVC gel actuator when the frequency ω = 1.04, 1.083 and 1.148, respectively.

Let $\lambda_{m}$ be the maximum amplitude when the PVC gel actuator resonates, and the corresponding frequency $\omega_{m}$ is the resonance frequency. It can be observed from Figure 12a that the PVC gel actuator has harmonic resonance when $\omega_{m}=1.083$, and the resonance peak $\lambda_{m}=4.35617$. At the same time, according to the Lyapunov exponent of Figure 12b, which is always less than zero, it can be seen that the PVC gel actuator has been in a stable vibration state, and no chaotic vibration has occurred. Therefore, dynamic responses, phase trajectories, and Poincaré maps corresponding to the harmonic resonance region are plotted to analyze the nonlinear dynamical characteristics of PVC gel actuators, as shown in Figure 13.

When the frequency ω=1.04, it can be seen from Figure 13a,d,g that the dynamic response is uniform and stable, the phase path presents a clear and stable “ring” shape, and the Poincaré map is a “radial” pattern. At this time, the vibration of the PVC gel actuator is in a stable multi-period motion state. When the frequency ω=1.083, as shown in Figure 13b,e,h, the amplitude λ gradually “beats” with the increase in time T and has a strong dynamic response. The points in the Poincaré map are irregularly scattered, and the phase path trajectory is clear and regular. From the Lyapunov exponent in Figure 12b, it can be seen that although strong resonance occurs at this time, there is a trend of chaotic vibration, but no chaotic vibration occurs. Therefore, it can be judged that the PVC gel actuator is a stable multi-period motion when harmonic resonance occurs. When the frequency ω=1.148, as shown in Figure 13c,f,i, the dynamic response amplitude λ decreases significantly at this time, and the amplitude of “beating” decreases continuously with the increase in time T. The phase path shows a clear “elliptical” trajectory, and the points of the Poincaré map show a “spiral” distribution. Therefore, the vibration of the PVC gel actuator is a relatively stable multi-period vibration state, and the vibration amplitude is more and more gentle. In summary, in the case of the damping effect, the PVC gel actuator has a trend of chaotic vibration at the resonance point in the whole harmonic resonance process. Still, the overall vibration is relatively stable, and the vibration amplitude also reaches the maximum at the resonance point and then decays rapidly.

**Figure 14:**
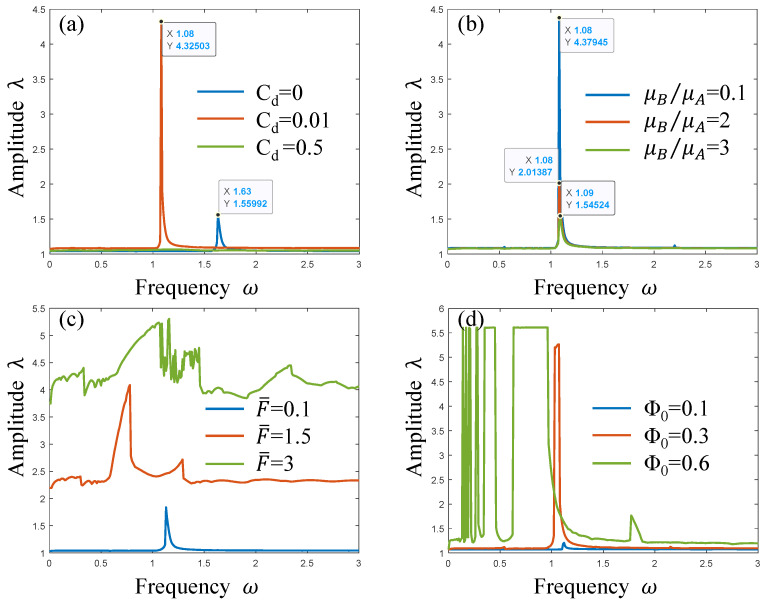
Amplitude–frequency characteristic diagram of viscous damping coefficient (**a**), shear modulus ratio (**b**), voltage amplitude (**c**), and prestress (**d**).

As shown in Figure 14, the amplitude–frequency characteristics of the PVC gel actuator are calculated according to the changes in different parameters. Figure 14a reflects the resonance change of the PVC gel actuator when the viscous damping coefficient $C_{d}=0,\;0.01\;\mathrm{and}\;0.5$. When $C_{d}=0$, the resonance frequency $\omega_{m}=1.63$ and the resonance peak $\lambda_{m}=1.55992$. When $C_{d}$ it increases from 0 to 0.01, the harmonic resonance amplitude $\lambda_{m}$ increases from 1.55992 to 4.32503, and the resonance interval shifts to the left; that is, the resonance frequency decreases. However, when $C_{d}=0.5$, the resonance is in a very small state and the vibration of the PVC gel actuator is in a stable state. Therefore, it can be seen that the existence of damping will change the resonance amplitude and resonance interval. The increase in damping will only reduce the amplitude and will not change the resonance interval. It can be seen from Figure 14b that as the ratio of shear modulus $\mu_{B}/\mu_{A}$ increases, the resonance frequency of the PVC gel actuator will not change, but the amplitude gradually decreases. In the process of $\mu_{B}/\mu_{A}$ increasing from 2 to 3, the amplitude is greatly reduced. It can be seen that when the ratio of shear modulus to $\mu_{B}/\mu_{A}$ is large, the resonance of the PVC gel actuator will be inhibited. As shown in Figure 14c, as the prestress $\bar{F}$ increases from 0.1 to 3, the resonance peak of the PVC gel actuator continues to increase, and the amplitude continues to rise. It can be seen that the increase in prestress will greatly improve the deformation of the PVC gel actuator. According to Figure 14d, when $\Phi_{0}=0.1$, the co-amplitude value is 1.38571, the voltage is low, and the vibration of the PVC gel actuator is weak. When $\Phi_{0}=0.3$, the amplitude reaches 1.44089. As the voltage continues to rise when $\Phi_{0}=0.6$, the resonance is disordered. It can be seen that the high voltage will cause the PVC gel actuator to lose effective resonance. It can be seen from the above content that the change of each parameter has an effect on the resonance amplitude of the PVC gel actuator. The prestress will change the resonance frequency, and the overload of the voltage will make the PVC gel actuator vibrate disorderly.

## 6. Bifurcation Characteristics and Lyapunov Exponent of PVC Gel Actuator Under Single Parameter Change

**Figure 15:**
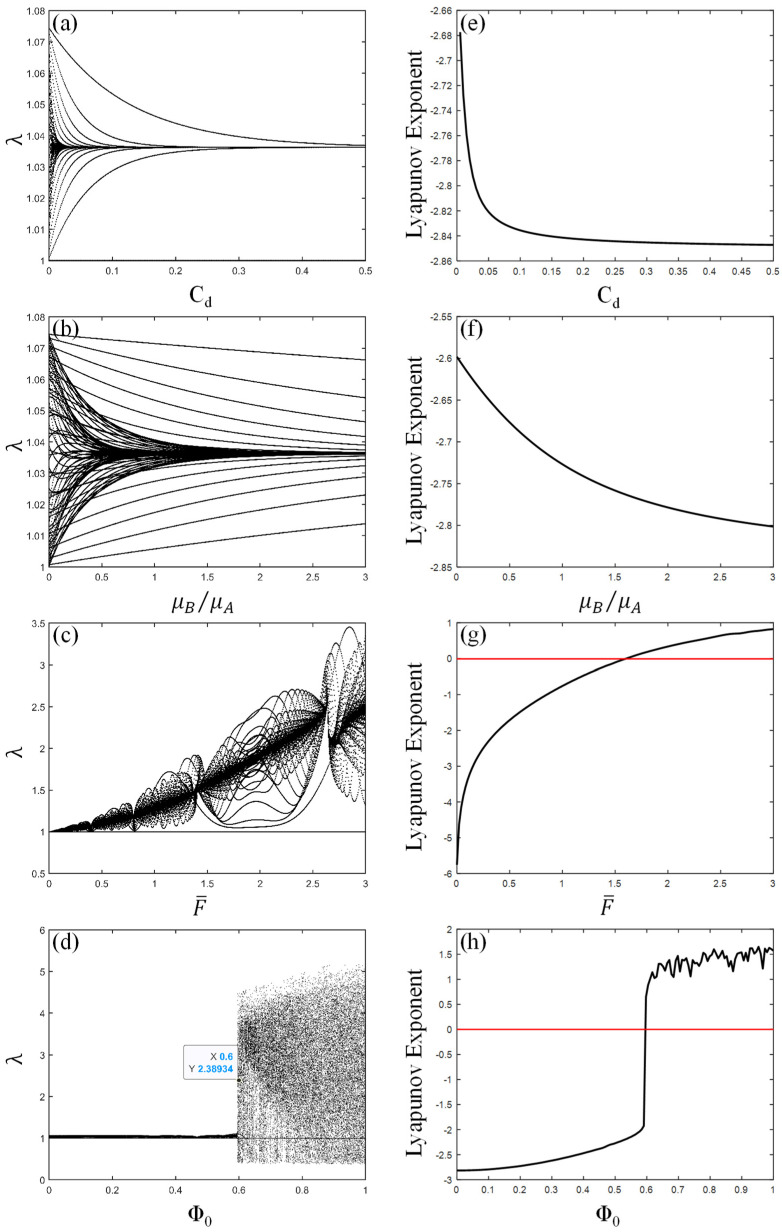
Bifurcation diagrams (**a**–**d**) and Lyapunov exponents (**e**–**h**) under different parameter changes. Viscous damping coefficient (**a**,**e**), shear modulus ratio (**b**,**f**), prestress (**c**,**g**), and voltage amplitude (**d**,**h**).

Figure 15 shows the rich bifurcation characteristics and Lyapunov exponent of PVC gel under electromechanical coupling with the change of viscous damping coefficient, shear modulus ratio, prestress and voltage amplitude. In Figure 15a, the vibration amplitude of the PVC gel actuator tends to be stable with the increase in damping. At the same time, it can be seen from the Lyapunov exponent shown in Figure 15h that the Lyapunov exponent value is always less than zero. Therefore, it can be judged that the periodicity of vibration of the PVC gel actuator increases gradually in the process of damping increase, and it is always in stable vibration without chaotic vibration. Figure 15b shows the vibration law of the PVC gel actuator with the change of shear modulus ratio $\mu_{B}/\mu_{A}$. With the increase $\mu_{B}/\mu_{A}$, the vibration of the PVC gel actuator gradually changes into multi-period vibration, and the larger the $\mu_{B}/\mu_{A}$, the smaller the period multiple. The Lyapunov exponent of Figure 15f is always less than zero, indicating that with the increase in the ratio of shear modulus, PVC gel is always in a stable vibration state, and no chaotic vibration occurs. As shown in Figure 15c, the vibration amplitude of the PVC gel actuator increases with the increase in prestress $\bar{F}$, and the periodic multiple is higher. The Lyapunov exponent in Figure 15g is greater than zero when the prestress $\bar{F}=1.5$, which indicates that the vibration of the PVC gel actuator is chaotic with the increase in prestress. As shown in Figure 15d, under a low voltage state, the PVC gel actuator is in quasi-periodic motion. With the increase in voltage amplitude at $\Phi_{0}=0.6$, the instantaneous vibration amplitude increases and transforms into chaotic motion, and the chaotic motion state continues with the increase in voltage amplitude. At the same time, the Lyapunov exponent of Figure 15h is greater than zero when $\Phi_{0}\geq 0.6$, which confirms the occurrence of chaos in the process of voltage increase in the PVC gel actuator. In summary, different parameter changes have different effects on the vibration state of the PVC gel actuator. The vibration of the PVC gel actuator under electromechanical coupling is affected by many factors.

## 7. Conclusions

In this study, the principle of elastic and viscous deformation of internal molecules of PVC gel was described by the SLS model, and the effective control equations of vibration deformation of PVC gel actuator were established by Gent model of hyperelastic material and work of voltage, prestress, inertial force and damping force. The dynamic response and phase path of the PVC gel actuator were obtained using the Runge–Kutta numerical algorithm, and the Poincaré maps were obtained using the stroboscopic method to analyze the periodicity of the vibration. At the same time, the stability of the vibration of the PVC gel actuator under the change of each parameter was quantitatively analyzed using the Lyapunov exponent. The results show that the vibration of the PVC gel actuator is always in a stable state with the increase in the ratio of shear modulus $\mu_{B}/\mu_{A}$ in the viscous damping coefficient $C_{d}$ and SLS model. The increase in prestress and voltage amplitude will cause a strong stress phenomenon of vibration, resulting in large amplitude chaotic vibration. 

According to the amplitude–frequency characteristics, the increase in damping will shift the resonance interval of PVC gel. The larger the ratio of shear modulus is, the smaller the amplitude is, and the resonance interval is unchanged. The increase in prestress will reduce the resonance frequency of the PVC gel actuator, and the amplitude will increase with the increase in prestress, but it will decrease with the decrease in prestress to a certain extent. The voltage will increase the co-amplitude value of the PVC gel actuator, but the overloaded voltage amplitude will cause resonance disorder of the PVC gel actuator. Finally, from the bifurcation diagram and Lyapunov exponent, it can be seen that the increase in the two electromechanical parameters of prestress and voltage to the critical condition will cause chaotic vibration of the PVC gel actuator. The theoretical results are consistent with the existing experimental studies. The theoretical research is suitable for applying PVC gel polymer, which can effectively control the deformation of PVC gel actuators through adjusting parameters. It can also be used for the deformation control of artificial soft tissue organs. The experimental study on the vibration of PVC gel film will be the focus of our forthcoming research.

## Data Availability

Data are contained within the article. Further inquiries can be directed to the corresponding author.
